# Effects of Precision Feeding on Economic and Productive Yields of Hyperprolific Lactating Sows Allocated at Different Farrowing Pens

**DOI:** 10.3390/ani15050763

**Published:** 2025-03-06

**Authors:** María Aparicio-Arnay, Natalia Yeste-Vizcaíno, Nerea Soria, Jorge Cambra, Beatriz Isabel, Carlos Piñeiro, Antonio Gonzalez-Bulnes

**Affiliations:** 1 Animal Data Analytics, S.L., C/Dámaso Alonso, 14, 40006 Segovia, Spain; carlos.pineiro@ada-animaldata.com; 2 Faculty of Veterinary Medicine, Universidad Complutense de Madrid (UCM), Ciudad Universitaria s/n, 28040 Madrid, Spain; bisabelr@ucm.es; 3 Cuarte S.L., Grupo Jorge, Ctra. de Logroño km 9.2, Monzalbarba, 50120 Zaragoza, Spain; nereasoria@cuartesa.com (N.S.); jorgecambra@cuartesa.com (J.C.); antoniogbulnes@cuartesa.com (A.G.-B.); 4 Faculty of Veterinary Medicine, Universidad Autónoma de Barcelona (UAB), 08193 Cerdanyola del Vallés, Spain; 5 Faculty of Veterinary Sciences, Universidad Cardenal Herrera—CEU, CEU Universities, C/Tirant lo Blanc, 7, 46115 Alfara del Patriarca, Spain

**Keywords:** farrowing pen, lactation, nutrition, precision feeding, swine

## Abstract

The results obtained using precision feeding in maternities (electronic sow feeders, ESFs) may be affected by the type of farrowing pen, as the design of the pen may modify the behavior of the sows. Hence, the aim of the present study was to determine the effects and possible interactions of electronic feeding systems in different types of farrowing pens (traditional, socialization and loose housing pens) on the productive and economic yields of lactating hyperprolific sows under commercial farm conditions. The results showed an overall lower feed disappearance in sows fed with ESFs and, consequently, a lower amount of feed per weaned piglet and per kg of weaned piglet, with evidence of improved welfare and no negative effects on body weight, condition at weaning or reproductive yields in the following cycle. These effects were, however, modulated by the type of pen and the type of bawl feeder; this finding highlights the need for improvement in the design of facilities and especially feeders to allow the sow a better use of available feed.

## Simple Summary

The aim of this study was to determine the effects of the electronic feeding systems used in different types of farrowing pens on the productive and economic yields of lactating hyperprolific sows under commercial farm conditions. The results indicate a remarkable decrease in feed wastage and, therefore, an improvement in the economic and environmental sustainability of production.

## 1. Introduction

The nutrition and housing of sows during farrowing and subsequent lactation are challenging aspects of swine production that dramatically affect the growth and viability of piglets and further productive yields of the sows. Moreover, there are extensive implications for both issues regarding animal welfare and the sustainability of the activity.

Adequate fulfillment of the nutritional requirements of sows during the different periods of their reproductive cycle, avoiding both under- and over-feeding, is critical and, consequently, is the focus of intense research [1,2,3]. At present, the implementation of precision farming—specifically, precision feeding (PF) based on the use of electronic sow feeders (ESFs)—constitutes a promising area of development. PF allows the feed intake of sows to be individually adjusted to provide an adequate feed amount, thus reducing feeding costs, nutrient wastage and environmental impacts [4,5]. Previous studies performed under farm conditions support the notion that ESFs allow the weaning of heavier piglets from sows with lower feed intake but better body condition than in traditional systems [6,7]. Hence, a lower amount of feed per kg of weaned piglet is used, resulting in a financial benefit of around EUR 0.5 per weaned piglet.

Interestingly, the feed consumption and consequent productive yields of sows are determined according to their behavior during lactation [8,9], which, in turn, is affected by housing. In brief, the feed intake is increased in females allowed and stimulated to move and stand.

Hence, based on different intake patterns, we can hypothesize that the design of the farrowing pen may affect the yields obtained using ESFs. The housing of lactating sows—specifically, the design of farrowing pens—is also a field of intense research and controversy in swine production. Traditionally, sows gave birth and suckled piglets in free-ranging pens. In the 1960s, the swine sector became more intensive, and the size of farms increased. Then, farrowing stalls were introduced to reduce space and costs, facilitate sow management and reduce piglet mortality by avoiding crushing during the first days after delivery. However, farrowing stalls isolate the female and, hence, restrict its innate behavior and diminish its welfare [10]. A plethora of studies evaluating the advantages/disadvantages of the system and possible alternatives (socialization crates and loose-house or free-ranging pens with or without temporary restriction) have been performed in the last few years (as reviewed by Pedersen et al. [11] and Goumon et al. [12]), given the technical issues and the growing concerns of society regarding animal restriction. Loose housing pens (supposedly the best option for animal welfare)—either with or without temporary confinement—are also the focus of intense debate. A recent, sophisticated review by Moustsen et al. [13] pointed out different problems related not only to animal welfare (loose housing may improve the welfare of the sows but decrease the welfare of the piglets, increasing their risk of wounds, fractures and death) but also to economic factors (cost efficiency) and environmental issues (increased food wastage and ammonia emissions in loose housing pens).

Because of these considerations, PF may be a robust tool for the accomplishment of adequate nutritional status, minimal food wastage and optimal cost efficiency in lactating sows. However, the design of the farrowing pen may affect the yields obtained when using ESFs. Hence, the aim of the present study was to determine the effects and possible interactions of using electronic feeding systems in different types of farrowing pens on the productive and economic yields of lactating hyperprolific sows under commercial farm conditions.

## 2. Materials and Methods

### 2.1. Ethics Statement

The research was carried out on a commercial farm (San Pedro, Plasencia de Jalón, Zaragoza, Spain) according to the European Union Directive and the Spanish Policy for Animal Protection RD53/2013. The Committee of Ethics in Animal Research of the Universidad Complutense de Madrid (UCM) assessed and approved the experimental procedures (CEEAH2788M2).

### 2.2. Animals, Management and Experimental Treatments

**Figure 1:**
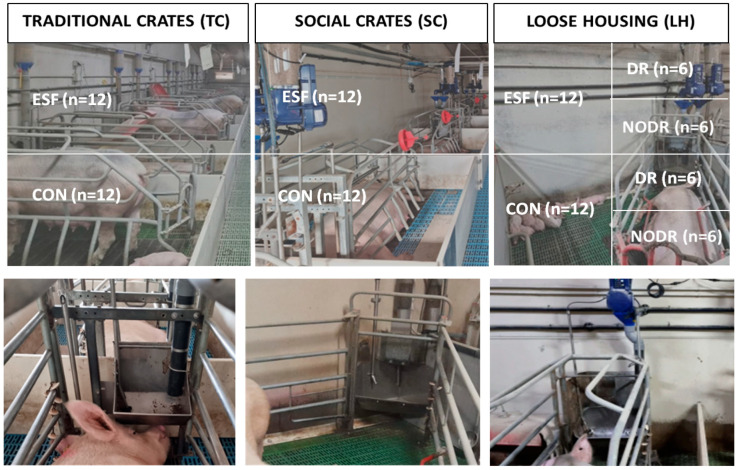
Schematic representation of the groups studied, with 12 sows fed with either electronic sow feeders (ESF group) or traditional feeders (CON group) in three different housing systems (traditional crates, TCs; social crates, SCs; and loose housing, LH; upper figure). Pictures of the bowl feeders are represented in the lower figure. Half of the sows in both the ESF and CON groups in the LH housing had bowl feeders with or without drainage (DR and NODR, respectively).

A total of 72 Danbred sows, from parity 2 to 7, were used. These sows were distributed in three different types of farrowing pens (Figure 1), considering their parity but also their condition score and historical productive data to ensure that females with similar features were allocated to each group: traditional crates (TCs; sows were facing the wall, *n* = 24), social crates (SCs; sows were allocated face to face in pairs, *n* = 24) and loose housing (LH; sows were released after five days of farrowing, *n* = 24) pens. The pen floors were the same in the three groups (plastic slats). From the week before parturition to the day of weaning (four weeks after delivery), half of the sows in each type of pen (*n* = 12) were fed with electronic sow feeders (ESF group; Gestal SOLO, Jyga Technologies Inc., Saint-Lambert-de-Lauzon, Quebec, Canada; six rations per day given at 05:00, 08:00, 11:00, 14:00, 17:00 and 20:00) while the remaining 12 sows were fed with traditional feeders (CON group; SB, Rotecna, Agramunt, Spain; two rations per day at 08:00 and 14:00).

The final bowl feeders were the same in all the pens and feeders in the social and traditional crates. However, the bowl feeders were larger and deeper in all the loose housing pens and were combined with drinking systems inside the bowls (outside the bowls in the TC and SC groups), and the farm workers reported water accumulation in some cases. Hence, to discern possible effects of such a design, drainage holes were employed in half of the LH feeders in both the ESF and CON groups so that we could differentiate four subgroups (LH-ESF-DR, LH-ESF-NODR, LH-CON-DR, LH-CON-NODR, *n* = 6 in each subgroup).

All sows were fed with the same standard grain-based diet (Ref. [14]; Supplementary Materials Table S1) following a reference feed intake basic curve. Such a feeding curve led to an increase in the amount of feed offered to the sows from the day of farrowing until day 11 post-farrowing, and a plateau was then established until the day of the weaning, after 24 days of lactation. However, the amount of feed served in the CON group could be adjusted manually by the staff of the farm depending on the feed disappearance of the previous day, while the amount of feed in the ESF group was automatically adjusted by the device, giving the sow the opportunity to eat 20% more feed each time.

At birth, productivity data were recorded for all the sows (Table 1). There were no significant differences in the number of total, alive and stillborn piglets or the mean litter weight at farrowing (recorded by using a litter scale; MPigData, Mensoft, Madrid, Spain) among sows allocated to the different farrowing crates and fed or not with ESFs.

After farrowing, all the piglets were sexed and individually identified using electronic eartags before, finally, within-group cross-fostering and allocation to mothers were performed at a rate of around 16 piglets/sow with similar birthweight. Creep feeding was offered to the piglets from 14 days old onward.

### 2.3. Feed Disappearance, Bodyweight and Condition, and Metabolic and Welfare Status

Feed disappearance was individually and daily recorded for each sow (manually in the CON group and automatically in the ESF group), from the day of farrowing to the day of weaning.

The weight and body condition (back-fat depth) of all the sows were measured at the beginning (i.e., one week before parturition) and at the end of the study (i.e., day of weaning 24 days after delivery). Body weight was measured using a dynamic (walk-through) scale in a corridor (Cima Control Pig, Correggio, Italy). Back-fat depth was measured at the P2 point, which lies on the right side of the animal 4 cm from the midline and transversal to the head of the last rib, using ultrasound equipment fitted to a multifrequency linear array probe (ProVetScan SF2 Wireless scanner, NewVetec, Leon, Spain). The initial and final bodyweight and back-fat depth of the sows were used to calculate losses in body weight and condition during lactation. The absolute body weight loss (ABWL) was calculated as the difference between weight after farrowing (initial weight before farrowing minus litter weight at birth) and weight at weaning, while the relative body weight loss (RBWL) was obtained by dividing the ABWL by the weight after farrowing, as previously described in other studies [15,16].

The metabolic status of all sows was assessed at the beginning of the study, at mid-lactation (day 14) and, after weaning, at day 28. Blood samples were drawn via puncture of the cranial vena cava (*cava cranialis*) using EDTA vacuum tubes (Vacutainer Systems Europe, Meylan-Cedex, France) and immediately centrifuged at 1500× *g* for 15 min (Nahita-Blue, Innovagen, Madrid, Spain). Plasma was immediately separated and stored at −80 °C until analyzed. Plasma concentrations of glucose (glucose and fructosamine), protein (total protein content) and lipids (total cholesterol, high- and low-density lipoprotein cholesterol [HDL-c and LDL-c, respectively] and triglycerides) were assessed with a clinical analyzer (Konelab 20i, Thermo Fisher Scientific, Madrid, Spain).

The welfare status of all sows was also assessed 14 days after farrowing and 3 days after weaning by assessing salivary biomarkers of stress (cortisol and alpha-amylase). Saliva samples were collected by introducing polypropylene sponges (Koronis ref. SKU 030004, La Griega E. Koronis, Madrid, Spain) clipped to flexible thin metal rods into each pig’s mouth to be chewed. Then, the sponges were placed into Salivette^®^ tubes (SARSTEDT S.A.U., La Roca del Vallès, Spain). The samples were immediately centrifuged at 3500× *g* for 10 min and stored at −80 °C until analyzed. Afterward, commercial assays were used for the quantification of salivary cortisol (Expanded Range High Sensitivity Salivary Cortisol Enzyme immunoassay kit, Salimetrics, Carlsbad, CA, USA) and alpha-amylase content (salivary alpha-amylase kinetic enzyme assay Kit, Salimetrics, Carlsbad, CA, USA [17]).

### 2.4. Productive and Economic Yields

Productive data, recorded per sow, included the total number of live and stillborn piglets at birth and total number of weaned piglets afterward. The total weight of the litter was recorded using a litter scale (MPigData, Mensoft, Madrid, Spain) at both farrowing and weaning. The daily records of maternal feed disappearance allowed the total feed disappearance during the lactation period and the kg of Feed per Weaned Piglet and per kg of Weaned Piglet to be determined. Afterward, the estimation of feed costs was based on the current price of lactation feed in the Spanish market (0.337 EUR/kg).

### 2.5. Productive and Reproductive Data in the Following Cycle

Data from the following farrowing were used to determine reproductive (interval from weaning to fertile estrus) and productive data (number of total piglets at birth).

### 2.6. Statistical Analysis

Data were analyzed using SPSS 22.0 (IBM, New York, NY, USA). The sow was considered the experimental unit. Two-way ANOVA and Duncan’s post hoc test were used to assess possible effects of the feeding system (CON vs. ESF) and the farrowing pen (TC vs. SC vs. LH) on feed disappearance, weight and metabolic and productive condition of sows. Changes over time were assessed by ANOVA for repeated measures (split-plot ANOVA). All results were expressed in the manuscript as mean ± SEM, with *p* < 0.05 representing statistical significance and 0.1 > *p* > 0.05 denoting a trend.

## 3. Results

### 3.1. Feed Disappearance

Overall, sows fed with electronic feeders showed significantly lower total feed disappearance during lactation than sows fed with traditional feeders (160.53 ± 3.05 kg in the ESF group vs. 183.62 ± 2.95 kg in the CON group; *p* < 0.001). This difference was found to be significantly lower when considering the type of crate in the ESF group with the traditional and socialization crates (TC and SC groups, *p* < 0.001 for both; Table 2). Conversely, despite a numerical difference, feed disappearance was statistically similar when comparing the ESF and CON groups in loose housing (LH group, *p* = 0.594) pens.

Feed disappearance in the LH pens was, however, significantly affected by the drainage of the feeder. Feed disappearance was similar in groups without drainage (163.18 ± 6.37 for ESF-NODR vs. 163.23 ± 6.42 for CON-NODR; *p* = 0.996). Drainage was related to increased feed disappearance (180.11 ± 5.37 in DR vs. 164.74 ± 5.63 in NODR; *p* = 0.063) and was numerically lower in ESF-DR feeders (176.34 ± 5.58 vs. 187.14 ± 5.88 for CON-DR; *p* = 0.200).

### 3.2. Bodyweight and Condition

There were no significant differences among sows allocated to the different types of crates in the mean bodyweight either at the beginning or the end of the trial. The assessment of bodyweight variation during lactation showed a higher ABWL in the SCs than in the TCs (−19.44 ± 3.26 vs. −6.93 ± 3.43; *p* = 0.032), which was only a trend when considering RBWL (−0.073 ± 0.013 in SCs vs. −0.027 ± 0.014; *p* = 0.05); the LH groups showed intermediate values. These sows in the SC group showed a higher back-fat depth at the beginning of the study (9.04 ± 0.56 mm) than sows in the TCs (6.45 ± 0.59 mm; *p* = 0.007) and LH pens (7.04 ± 0.59 mm; *p* = 0.05). There were no differences among the types of crates at weaning.

The assessment of the type of feeder showed that, overall, mean bodyweight and back-fat depth at both the beginning and the end of the experimental trial, as well as the ABWL and RBWL, were similar between sows fed with electronic sow feeders and traditional feeders. No significant differences in these parameters were found among ESF and CON sows in the different crates (Table 3).

We note significant differences between subgroups with and without drainage in the LH crates (DR and NODR, respectively). There were no significant differences in bodyweight loss, but sows in the DR subgroup showed a trend of increasing backfat depth, while sows in the NODR lost backfat (*p* = 0.06); this effect was similar to that of sows in the CON and ESF groups.

### 3.3. Metabolic and Welfare Status

The assessment of salivary cortisol at both 14 days after farrowing and 3 days after weaning showed no differences among feeders and pens (Table 4).

Conversely, 14 days after farrowing, the concentration of alpha-amylase was significantly lower in the sows of the ESF group (69.12 ± 29.31 U/L) than in sows of the CON group (208.68 ± 28.33; *p* = 0.001). There were also significant effects of the type of crate, with a higher concentration of alpha-amylase in sows in the SC group (234.82 ± 33.60 U/L) than in LH (68.22 ± 35.96 U/L; *p* = 0.004) and TCs (109.16 ± 36.29 U/L; *p* = 0.041). Concomitantly, the assessment of the ESF and CON groups in SCs showed significant differences (59.17 ± 61.67 vs. 410.47 ± 61.67 U/L, respectively; *p* < 0.001). After weaning, the high values in the CON group in SCs showed a significant decrease (*p* = 0.014), which was not found in the other groups. Hence, there were no significant differences among types of feeders and pens during lactation.

The analysis of mean plasma concentrations for the indexes of glucose, lipids and protein metabolism (Supplementary Table S2) showed similar values between the CON and ESF groups at all points of the study, except for significantly higher cholesterol and HDL concentrations at weaning in the ESF group (*p* < 0.05 for both). An interaction was found between the type of feeder and pen at mid-lactation, in that blood concentrations of fructosamine were higher in the ESF sows in the TCs (379.37 ± 15.31 vs. 340.93 ± 7.48; *p* = 0.044), and at weaning, in that higher concentrations of cholesterol, glucose and HDL were found in the ESF sows allocated to SCs (*p* < 0.05 for all).

### 3.4. Productive Data at Weaning and Economic Yields

There were no significant differences in the number of weaned piglets and the litter weight at weaning among the different types of crates and feeders (Table 5). This was as expected due to the management procedures for hyperprolific sows (within-group cross-fostering maintaining similar litter weight and size and creep feeding from 14 days old onward).

However, when determining the cost of rearing such litters, significant differences were found between types of feeders but not among types of crates. There were overall, significant differences between the ESF and CON groups in terms of kg of Feed per Weaned Piglet (11.59 ± 0.38 vs. 13.20 ± 0.36, respectively; *p* = 0.003) and kg of Feed per kg of Weaned Piglet (2.03 ± 0.08 vs. 2.43 ± 0.07; *p* < 0.001). Such significant differences were confirmed when analyzing sows allocated to TCs and SCs separately but, conversely, were not found for the LH pens (Table 5).

Conversely, significant differences were found between sows with drainage systems in their feeders in the LH pens. With a similar number of weaned piglets and litter weight, the sows with drainage systems in their pens exhibited increased kg of Feed per Weaned Piglet (13.58 ± 0.61 vs. 11.48 ± 0.65 for NODR; *p* = 0.029) and tended to show increased kg of Feed per kg of Weaned Piglet (2.54 ± 0.13 vs. 2.18 ± 0.14 for NODR; *p* = 0.074). Moreover, the presence or not of the drainage system affected the yields obtained with the ESFs. There was a similar kg of Feed per kg of Weaned Piglet for the sows in the ESF and CON groups without drainage (2.10 ± 0.10 vs. 2.14 ± 0.10 in the CON group; *p* = 0.815). However, when drainage was performed, the kg of Feed per kg of Weaned Piglet was significantly lower in the ESF sows than the CON sows (2.34 ± 0.18 vs. 3.44 ± 0.19 in the CON group; *p* < 0.001).

### 3.5. Productive Data in Following Reproductive Cycle

There were no significant effects of the different types of pens and feeders used during the studied cycle on the reproductive parameters of the following cycle; i.e., the intervals of weaning to estrus (WEI) or between farrowing (FFI) and the number of total piglets. WEI in the ESF sows was 5.25 ± 0.71 days vs. 5.74 ± 0.83 days in the CON (*p* = 0.679), while FFI was 148.19 ± 2.04 and 147.46 ± 2.14 days for ESF and CON groups, respectively (*p* = 0.370). The values for WEI and FFI were also similar among the different types of pens (147.41 ± 2.49, 148.36 ± 2.06 and 147.59 ± 2.23 days for the SC, LH and TC groups, respectively). Concomitantly, there were no significant differences in the number of total piglets in the following cycle between feeders (20.93 ± 1.69 and 20.57 ± 1.29 for the ESF and CON groups, respectively) or pens (20.79 ± 1.48, 21.78 ± 1.29 and 19.35 ± 1.60 for SCs, LH and TCs, respectively).

## 4. Discussion

The results of the present study showed that the use of precision feeding improves the productive and economic yields of hyperprolific lactating sows by reducing feed disappearance without penalizing the body condition of the sows and the number and weight of weaned piglets. Consequently, the cost of weaned piglets (either considering the cost per kg of piglet or the total cost of piglet) was significantly decreased. However, such an effect was modulated by the type of farrowing pen as well as by the type of final bowl feeder, which highlights the critical importance of facilities.

The lower feed disappearance and cost of producing piglets when using ESFs were, however, significantly affected by the type of pen. Differences in feed disappearance were higher and significantly different when comparing sows housed in traditional crates (TC group; 29.2 kg less in ESFs, meaning 0.79 EUR per weaned piglet and around EUR 23,600 per year in a farm with 1000 breeding sows) and even more in socialization crates (SC group; 35.5 kg less in ESFs, meaning 0.96 EUR per weaned piglet and around EUR 28,700 per year in a farm with 1000 breeding sows).

Conversely, the differences in sows in loose-housing pens were lower and did not reach statistical significance (LH group; 4.6 kg less in ESFs) because, when compared with sows in other pens, ESF sows exhibited increased feed disappearance at around 15 kg, and CON sows showed decreased feed disappearance at around 10 kg. The lower efficiency of the ESF system in the LH pens was, however, found to be influenced by the type of bowl feeder. Feed disappearance was almost equal in the ESF and CON groups without drainage holes, but lower for ESFs with drainage (around 11 kg less in ESFs, meaning 0.30 EUR per weaned piglet and around EUR 8900 per year in a farm with 1000 breeding sows). The causes of these findings may be related to the accumulation of water and highlight the need for the adequate design of facilities in either case but mainly when applying precision systems.

Firstly, it is known that a feeder with a large amount of water impedes easy access of the sow to the feed, causes play behavior and water wastage and compromises adequate feed intake because feed is diluted in liters of water and the sow gets bored of drinking without being able to reach the feed [18,19,20]. The authors [19,20] also report significant improvements in feed consumption, production efficiency and litter performance when sows have the freedom to decide the time and amount of eating and drinking.

Secondly, the accumulation of water causes fermentation of the feed. The water accumulated inside the feeder mixed with feed is prone to microbial contamination and fermentation because of the warm temperature inside the lactation barn [21]. Sows usually refuse to consume such water and feed [22], and farmers must frequently remove the wastage from the bowl. In a recent study, Tajudeen and coworkers [23] estimated that the availability of dry feed and fresh water increases feed intake by around 9%, which is exactly the same as the increase found in our study after implementing drainage holes.

A higher feed consumption should favor the body condition and the metabolic and welfare status of the sow. Previous authors described an effect of the feeding pattern wherein a higher number of meals increased the feed intake (0.5 kg/d) and resulted in a 1.8 kg lower body weight loss during a 26-day lactation period [24]. Conversely, in our study, no significant effects of the feeding system or the pen on body weight loss or backfat depth variation during lactation were found. However, we need to bear in mind that we did not measure the feed intake but feed disappearance.

Conversely, the assessment of metabolic features showed that, overall, ESFs increased concentrations of glucose, fructosamine and cholesterol, mainly in SCs. Moreover, the welfare of the sows fed using ESFs was higher and had a strong interaction with the type of pen. There were no differences when salivary cortisol was assessed, but salivary alpha-amylase, a robust biomarker of stress in pigs [25,26,27], was significantly lower in sows fed with ESFs, mainly in the SC pens again. Such findings support previous studies addressing multifactorial causes of stress and the fact that the same stressor may induce different stress responses [28]. From our data, we can hypothesize that the SCs favor the interaction among sows in the room but, concomitantly, such an interaction may favor the spread of feeding mimicking and stress responses among the individuals in the same room. In any case, these findings on the effect of the type of pen and size and drainage of feeders support the need for adequate facilities and good management with optimal nutritional and welfare conditions to ensure productive yields [29,30,31].

In summary, the sows in ESFs, despite their lower feed disappearance, had similar or even better metabolic and welfare status and were weaned with a similar bodyweight and back fat as sows fed with CON feeders. Furthermore, there were no differences in the reproductive features in the following cycle. Such a finding suggests a similar feed intake, sustaining differences in feed wastage, between the groups of the current study since, although changes in body condition typically observed in sows housed in commercial production units are too slight to have an effect on reproductive performance [32], reduced feed intake results in excessive weight and condition loss which, in turn, causes a lower percentage of sows returning to estrus within 10 days of weaning. This also leads to extended mating intervals, lower pregnancy rates and smaller subsequent litters [33].

Hence, if homeostasis, body condition and subsequent reproductive performance of sows were not impaired by a supposed lower feed intake, we can hypothesize that the difference was, in fact, a lower feed wastage when using ESFs. This is economically remarkable since it leads to savings of around EUR 7.8 per lactating sow, which, in turn, means around EUR 18,600 per year in a farm with 1000 sows and is beneficial in terms of environmental sustainability.

In this way, our present study shows that precision feeding in lactating sows offers a significant means of achieving sustainable swine production. Reducing feed disappearance through individualized feed allocation via ESFs minimizes resource misuse and lowers the environmental impact associated with feed production and disposal [34] while also reducing the nitrogen intake by 10–13% and total phosphorus intake by 6–9% as demonstrated by Cloutier et al. in another study in gestating sows [35]. Hence, while the study demonstrated economic benefits, the environmental implications extend beyond financial gains. Reduced feed disappearance while maintaining good performance directly translates to decreased greenhouse gas emissions (in terms of CO_2_ equivalents) from feed production and manure management [36]. Furthermore, the ESF system’s potential to optimize sow health and welfare, though requiring careful attention to facility design (e.g., addressing water accumulation issues in loose housing pens in our study), contributes to more sustainable and ethically sound farming practice. In summary, integrating technological advancements with careful consideration of animal welfare and environmental sustainability is crucial for successful and responsible livestock production.

## 5. Conclusions

The results of this study indicated that the use of precision feeding on farms of hyperprolific sows results in a remarkable decrease in feed disappearance and, therefore, in the economic and environmental sustainability of production. Such advantages are boosted by improving the design of the facilities and especially the feeders to allow the sow a better use of available feed.

## Figures and Tables

**Table 1:** Mean ± S.E.M. number of piglets in each litter and mean litter weight in sows allocated to traditional crates (TCs), socialization crates (SCs), or loose housing (LH) pens, and fed with electronic sow feeders (ESFs) or traditional (CON) feeders.

Group		Total Piglets	Alive Piglets	Stillborn Piglets	Litter Weight (kg)
**TC**	**ESF**	23.00 ± 1.33	19.33 ± 1.19	3.67 ± 0.63	23.67 ± 1.20
	**CON**	23.25 ± 1.16	20.71 ± 1.03	2.50 ± 0.73	23.57 ± 1.04
	*p*-value	0.889	0.380	0.242	0.954
**SC**	**ESF**	19.50 ± 1.64	18.33 ± 1.48	1.17 ± 0.45	24.86 ± 1.12
	**CON**	19.92 ± 1.64	18.58 ± 1.48	1.33 ± 0.45	25.28 ± 1.12
	*p*-value	0.859	0.906	0.797	0.795
**LH**	**ESF**	22.91 ± 0.92	20.09 ± 0.85	2.82 ± 0.70	25.93 ± 1.05
	**CON**	21.90 ± 0.97	18.00 ± 0.89	3.90 ± 0.74	22.89 ± 1.10
	*p*-value	0.460	0.106	0.300	0.060

**Table 2:** Mean ± S.E.M. of feed disappearance in sows allocated to traditional crates (TCs), socialization crates (SCs), or loose housing (LH) pens and fed with electronic sow feeders (ESFs) or traditional (CON) feeders.

Group		Feed Disappearance (kg)
**TC**	**ESF**	156.51 ± 5.34
	**CON**	185.68 ± 4.63
	*p*-value	<0.001
**SC**	**ESF**	154.52 ± 4.58
	**CON**	189.99 ± 4.58
	*p*-value	<0.001
**LH**	**ESF**	170.60 ± 5.85
	**CON**	175.20 ± 6.14
	*p*-value	0.594

**Table 3:** Mean ± S.E.M. bodyweight before farrowing minus litter weight (BWLW, kg) back-fat depth before farrowing (BFDBF; mm), bodyweight at weaning (BW, kg), back-fat depth at weaning (BFD, mm) and absolute and relative bodyweight loss (ABWL and RBWL, respectively) in sows allocated to traditional crates (TCs), socialization crates (SCs), or loose housing (LH) pens and fed with electronic sow feeders (ESFs) or traditional (CON) feeders.

GROUP				Weaning		Weight Loss Variation	
		BWLW	BFDBF	BW	BFD	ABWL	RBWL
**TC**	**ESF**	231.22 ± 10.65	7.12 ± 0.70	227.78 ± 9.18	7.34 ± 0.65	−3.45 ± 3.74	−0.01 ± 0.016
	**CON**	230.59 ± 9.22	5.78 ± 0.60	223.00 ± 8.31	7.50 ± 00.59	−10.42 ± 3.39	−0.04 ± 0.014
	*p*-value	0.965	0.162	0.704	0.861	0.184	0.151
**SC**	**ESF**	242.31 ± 17.93	9.09 ± 0.90	225.25 ± 7.07	7.90 ± 0.70	−17.06 ± 5.18	−0.07 ± 0.028
	**CON**	218.89 ± 17.93	8.99 ± 0.94	221.00 ± 7.29	9.25 ± 0.73	−21.82 ± 5.68	−0.08 ± 0.020
	*p*-value	0.309	0.942	0.682	0.195	0.542	0.701
**LH**	**ESF**	233.07 ± 7.43	6.42 ± 0.81	219.00 ± 7.27	7.57 ± 0.79	−14.07 ± 4.66	−0.06 ± 0.019
	**CON**	232.71 ± 7.79	7.66 ± 0.85	214.40 ± 7.63	8.13 ± 0.83	−18.31 ± 4.88	−0.08 ± 0.020
	*p*-value	0.974	0.304	0.667	0.632	0.537	0.473

**Table 4:** Mean ± S.E.M. cortisol and alpha-amylase concentration of sows allocated to traditional crates (TCs), socialization crates (SCs), or loose housing (LH) pens and fed with electronic sow feeders (ESFs) or traditional (CON) feeders.

GROUP		14 Days After Farrowing		3 Days After Weaning	
		Cortisol (μg/dL)	Alpha-Amylase (U/L)	Cortisol (μg/dL)	Alpha-Amylase (U/L)
**TC**	**ESF**	0.039 ± 0.86	90.54 ± 55.41	0.024 ± 0.006	203.30 ± 96.04
	**CON**	0.041 ± 0.01	127.78 ± 47.99	0.020 ± 0.005	202.51 ± 81.90
	*p*-value	0.855	0.617	0.558	0.995
**SC**	**ESF**	0.059 ± 0.01	59.17 ± 61.67	0.090 ± 0.04	65.61 ± 38.59
	**CON**	0.052 ± 0.01	410.47 ± 61.67	0.052 ± 0.04	126.48 ± 41.27
	*p*-value	0.609	<0.001	0.472	0.300
**LH**	**ESF**	0.042 ± 0.01	57.64 ± 21.46	0.019 ± 0.004	185.37 ± 72.47
	**CON**	0.053 ± 0.01	78.80 ± 22.51	0.017 ± 0.005	98.54 ± 80.12
	*p*-value	0.501	0.505	0.756	0.432

**Table 5:** Mean ± S.E.M. number of weaned piglets and total litter weight in sows allocated to traditional crates (TCs), socialization crates (SCs), or loose housing (LH) pens and fed with electronic sow feeders (ESFs) or traditional (CON) feeders.

Group		Weaned Piglets (n)	Litter Weight at Weaning (kg)	Kg of Feed per Kg of Weaned Piglet	Kg Feed per Weaned Piglet
**TC**	**ESF**	14.78 ± 0.36	75.04 ± 1.67	1.93 ± 0.10	10.76 ± 0.61
	**CON**	14.58 ± 0.31	79.88 ± 1.67	2.38 ± 0.09	12.78 ± 0.53
	*p*-value	0.690	0.110	0.004	0.022
**SC**	**ESF**	13.33 ± 0.50	86.81 ± 6.86	1.87 ± 0.14	11.73 ± 0.62
	**CON**	12.92 ± 0.50	74.59 ± 6.86	2.49 ± 0.14	13.92 ± 0.62
	*p*-value	0.417	0.255	0.006	0.021
**LH**	**ESF**	14.18 ± 0.50	76.60 ± 5.78	2.30 ± 0.14	12.28 ± 0.69
	**CON**	13.70 ± 0.52	61.84 ± 6.10	2.44 ± 0.15	12.91 ± 0.73
	*p*-value	0.512	0.154	0.521	0.536

## Data Availability

All data are included in the manuscript and Supplementary Tables S1 and S2.

## Supplementary Materials

The following supporting information can be downloaded at: https://www.mdpi.com/article/10.3390/ani15050763/s1, Table S1: Composition and calculated analysis of the diet used in the experimental procedure. Table S2: Plasma concentrations for parameters of glucose, lipids and protein metabolism upon allocation in pens, mid-lactation and weaning in sows allocated to traditional crates (TCs), socialization crates (SCs), or loose housing (LH) pens and fed with electronic sow feeders (ESFs) or traditional (CON) feeders.
